# A large arteriovenous fistula steals a considerable part of systemic blood flow during veno-arterial extracorporeal circulation support in a porcine model

**DOI:** 10.3389/fphys.2023.1109524

**Published:** 2023-07-11

**Authors:** A. Valerianova, M. Mlcek, O. Kittnar, T. Grus, L. Tejkl, V. Lejsek, J. Malik

**Affiliations:** ^1^ Third Department of Internal Medicine, First Faculty of Medicine, General University Hospital in Prague, Charles University in Prague, Prague, Czechia; ^2^ First Faculty of Medicine, Institute of Physiology, Charles University in Prague, Prague, Czechia; ^3^ Second Surgical Clinic—Cardiovascular Surgery, First Faculty of Medicine, General University Hospital in Prague, Charles University in Prague, Prague, Czechia

**Keywords:** animal model, veno-arterial extracorporeal membrane oxygenation, arteriovenous fistula, cerebral blood flow, cerebral tissue oxygenation

## Abstract

**Background:** Veno-arterial extracorporeal membrane oxygenation (V-A ECMO) is one of the most frequently used mechanical circulatory support devices. Distribution of extracorporeal membrane oxygenation flow depends (similarly as the cardiac output distribution) on regional vascular resistance. Arteriovenous fistulas (AVFs), used frequently as hemodialysis access, represent a low-resistant circuit which steals part of the systemic perfusion. We tested the hypothesis that the presence of a large Arteriovenous fistulas significantly changes organ perfusion during a partial and a full Veno-arterial extracorporeal membrane oxygenation support.

**Methods:** The protocol was performed on domestic female pigs held under general anesthesia. Cannulas for Veno-arterial extracorporeal membrane oxygenation were inserted into femoral artery and vein. The Arteriovenous fistulas was created using another two high-diameter extracorporeal membrane oxygenation cannulas inserted in the contralateral femoral artery and vein. Catheters, flow probes, flow wires and other sensors were placed for continuous monitoring of haemodynamics and organ perfusion. A stepwise increase in extracorporeal membrane oxygenation flow was considered under beating heart and ventricular fibrillation (VF) with closed and opened Arteriovenous fistulas.

**Results:** Opening of a large Arteriovenous fistulas (blood flow ranging from 1.1 to 2.2 L/min) resulted in decrease of effective systemic blood flow by 17%–30% (*p* < 0.01 for all steps). This led to a significant decrease of carotid artery flow (ranging from 13% to 25% after Arteriovenous fistulas opening) following VF and under partial extracorporeal membrane oxygenation support. Cerebral tissue oxygenation measured by near infrared spectroscopy also decreased significantly in all steps. These changes occurred even with maintained perfusion pressure. Changes in coronary artery flow were driven by changes in the native cardiac output.

**Conclusion:** A large arteriovenous fistula can completely counteract Veno-arterial extracorporeal membrane oxygenation support unless maximal extracorporeal membrane oxygenation flow is applied. Cerebral blood flow and oxygenation are mainly compromised by the effect of the Arteriovenous fistulas. These effects could influence brain function in patients with Arteriovenous fistulas on Veno-arterial extracorporeal membrane oxygenation.

## Introduction

Veno-arterial extracorporeal membrane oxygenation (V-A ECMO) is one of the most frequently used mechanical circulatory support devices (Vahdatpour, Collins et al., 2019). Indications for V-A ECMO according to current guidelines (McDonagh, Metra et al., 2021; Soar, Bottiger et al., 2021) include severe heart failure, cardiogenic shock, or refractory cardiac arrest. The main aim of mechanical circulatory support use is to maintain organ perfusion during these critical states.

Arteriovenous fistulas (AVF) represent a low-resistance circuit, which steals part of the cardiac output. They are created intentionally as a vascular access for hemodialysis, or could be formed as a complication of trauma, catheterization, etc. To describe the impact of AVFs on systemic perfusion, the term effective cardiac output (COeff) is used–and is calculated as cardiac output minus AVF flow (Amerling, Ronco et al., 2011; Basile and Lomonte, 2018). We know from human studies that the presence of a high-flow AVF (with AVF flow over 2 L/min or with flow representing over 30% of cardiac output (Reddy, Melenovsky et al., 2016)) substantially decreases COeff and compromises cerebral tissue oxygenation (Malik, Valerianova et al., 2021). Competition of AVF and other low-resistant vascular beds was also documented in our animal model of high-flow AVF circulation (Valerianova, Mlcek et al., 2022). It is known that the circulation partly or fully dependent on VA-ECMO or on other extracorporeal life-supporting devices, for example, during on-pump cardiac surgery, differs considerably from normal circulation. The main difference is its non-pulsatile flow. The flow distribution depends on the regional vascular resistance and the presence of AVF could change blood delivery to various (vital) organs.

We hypothesized that the presence of a high-flow AVF significantly decreases organ perfusion in both partial and full V-A ECMO support. Therefore, we designed a study in porcine biomodel focused on haemodynamics and blood flow distribution during V-A ECMO support with and without large AVF. The project was intended to study the circulatory changes in healthy heart conditions.

## Materials and methods

The study was performed in The Common Experimental Laboratory of the Department of Physiology, First Faculty of Medicine, Charles University, Prague, on female domestic pigs (*Sus scrofa domestica*). The animals were handled in accordance with guidelines and legal requirements for animal use in research. The study was approved by Institutional care and use committee and was performed in accordance with European Guidelines on Laboratory Animal Care. The number of animals 6) was based on our previous study that used the same model (Valerianova, Mlcek et al., 2022) and on the RRR principle (replacement, reduction, refinement) of animal research.

### The protocol

The experiments were performed on female domestic swine (*Sus scrofa domestica*). All the experiments were held under general anesthesia. After premedication with intramuscular (i. m.) injection of midazolam (0.3 mg/kg) and ketamine (20 mg/kg), the marginal ear vein was cannulated. After preoxygenation with 100% oxygen via a facial mask, the general anesthesia was induced with intravenous bolus (i.v.) of propofol (1–2 mg/kg) followed by a continuous infusion. The orotracheal intubation was performed and mechanical ventilation was started. During the procedure, the ventilation was adjusted to maintain normoxia (peripheral oxygen saturation ≥97%, pO_2_ 100 mmHg) and normocapnia (EtCO_2_ 38–40 mmHg, pCO_2_ 35–45 mmHg). During the whole experiment total intravenous anesthesia was maintained by continuous intravenous infusion of propofol, morphine and midazolam with regular controls of pupillary and corneal reflexes to adjust the depth of anesthesia. Doses of anesthetics remained unchanged during the protocol to avoid the effect of dose variation on brain perfusion and cardiovascular system. Anticoagulation was maintained by unfractionated heparin, starting with i. v. bolus 100 units per kilogram followed by continuous i. v. infusion to reach the target activated clotting time (200–250 s). Initial fluid bolus of 1000 mL was followed by continuous i. v. infusion to maintain central venous pressure between 6–8 mmHg. No inotropes or vasopressors were used during the experiment.

Sheaths and catheters were inserted into the jugular vein and the brachial arteries; measurement probes and catheters were inserted as described in section *Measured parameters*.

**FIGURE 1:**
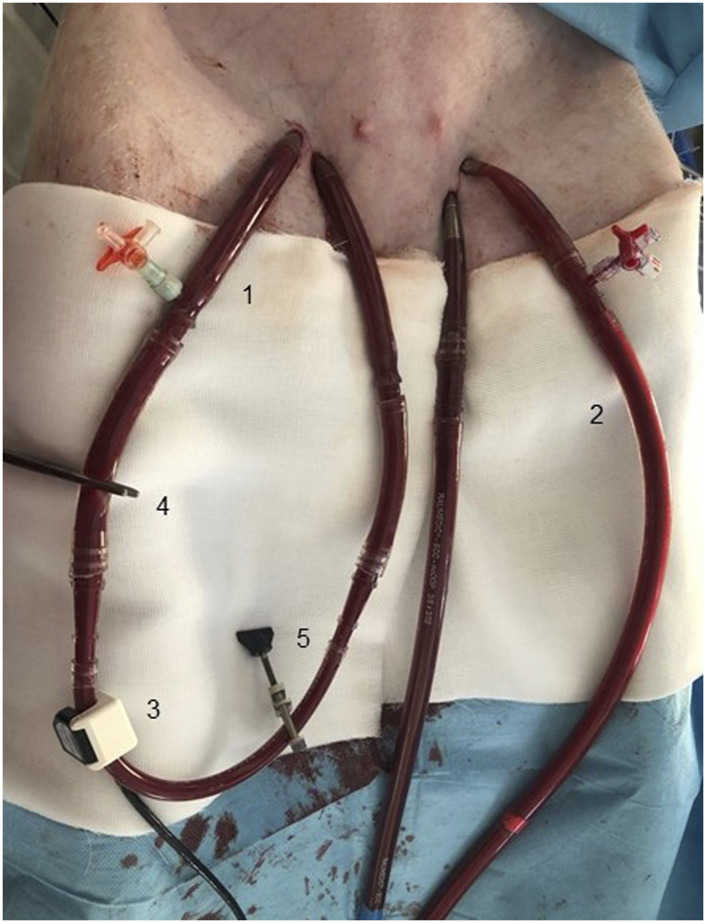
Insertion of ECMO cannulas for V-A ECMO support and to create an AVF. Arteriovenous fistula was created using two ECMO cannulas connected by a plastic tube 1). Two cannulas inserted in contralateral femoral vessels were used for connection to the ECMO circuit 2). AVF and ECMO flow were measured using an ultrasound probe 3). Hemostatic forceps was used to close/open the arteriovenous fistula 4). AVF blood flow was regulated using a special clamp 5).

Standard 18F and 23F ECMO cannulas were inserted into the ipsilateral femoral vessels percutaneously under imaging intensifier guidance. The tip of the venous cannula was advanced into the inferior vena cava and placed close to the right atrium. The tip of the arterial cannula was advanced into the descending thoracic aorta and placed close to the aortic arch. ECMO blood flow was continuously measured by transient-time ultrasound probe fixed to the circuit. AVF was created by connection of contralateral femoral artery and femoral vein using other percutaneously inserted shorter ECMO cannulas (18 F for the arterial cannula and 22 F for venous cannula) as described previously (Valerianova, Mlcek et al., 2022), see Figure 1. The tips of AVF cannulas were located in the common iliac artery or vein, or closely after aortic/inferior vena cava bifurcation. AVF blood flow was continuously measured by transient-time ultrasound probe fixed to the ECMO set (Transonic, USA). A special clamp fixed around the arterial cannula was used to regulate the AVF blood flow and to close the fistula when required, see Figure 1. Initial AVF flow measurement was performed to verify a sufficient AVF functioning. Ventricular fibrillation was induced by rapid right ventricular pacing using a stimulation electrode inserted via the right jugular vein.

**FIGURE 2:**
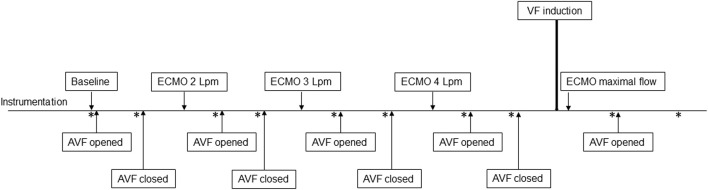
Scheme of study protocol. Measurements were performed in baseline, and after 20 min of stabilization after changing ECMO flow or opening the AVF. Points of measurement are marked with an asterisk.

The protocol is summarized in Figure 2. Baseline parameters with and without AVF were recorded with ECMO flow set on 500 mL/min to prevent clotting of the circuit. According to our experience with this high-flow AVF animal model (Valerianova, Mlcek et al., 2022), hemodynamic parameters stabilize in approximately 15 min after AVF opening. Based on these results the measurements were made in 20th minute of each step. After establishing the model, the following protocol steps were analyzed: Baseline with AVF closed/opened, ECMO flow 2 L/min with AVF closed/opened, ECMO flow 3 L/min with AVF closed/opened, ECMO flow 4 L/min with AVF closed/opened, VF with maximal ECMO flow with AVF closed/opened. VF data is presented in a separate paragraph. After completing the protocol, the animals were euthanized by intravenous injection of potassium chloride and termination of ECMO support.

### Measured parameters

Continuous surface ECG, peripheral oxygen saturation and EtCO_2_ monitoring were performed. Continuous invasive arterial blood pressure was measured using a monitoring catheter inserted in the left radial artery. Central venous pressure was measured using the central venous catheter inserted via the left jugular vein. Pulmonary arterial pressure, continuous measurement of mixed venous oximetry (SvO_2_) and cardiac output measured by thermodilution method were recorded using the Swan-Ganz catheter (CCOmbo, Edwards Lifesciences) inserted deep into the pulmonary artery control via the right jugular vein under imaging intensifier guidance. Carotid artery flow was continuously measured by transient-time perivascular ultrasound probe (Transonic, United States). The intracoronary artery flow velocity was measured by a Doppler flow wire (FloWire, Volcano, USA) inserted into the left anterior descending or circumflex artery approximately 8 cm distal to the left coronary artery ostium. Measurement of regional tissue oxygen saturation was performed using the INVOS 5100C oximetry system (Medtronic), working on the near-infrared spectroscopy principle. The sensors were placed as follows; 1. on the front leg; 2. on the top of the head; 3. on the back and 4. on the hind leg with the arteriovenous fistula. Invasive tissue blood microflow measurement was performed by laser-Doppler tissue blood flow monitor OxyFloTM (Oxford Optronix, United Kingdom) with probes placed in both cerebral frontal lobes and on the tongue. Tissue partial pressure of oxygen was recorded by fibre-optic oxygen micro-sensor technology, OxyLiteTM (Oxford Optronix, United Kingdom), with sensors inserted into cerebral frontal lobes and liver.

### Calculated parameters

Mean arterial pressure (MAP) was calculated as MAP = 2/3*ART(d) + 1/3*ART(s) (ART–systemic arterial pressure, d)—diastolic, s)—systolic). Stroke volume (SV) was calculated as SV = CO/HR (CO–cardiac output, HR–heart rate). Total flow (TF) was calculated as cardiac output plus ECMO flow. Effective total flow (TFeff) vas calculated as TF minus AVF flow. Left ventricular stroke work was approximated as *SVx(MAP-PCW)x0.0136* (SV–stroke volume, MAP–mean arterial pressure, PCW–pulmonary capillary wedge pressure). In analogy, right ventricular stroke work was calculated as *SVx(PAMP-CVP)x0.0136* (PAM–pulmonary artery mean pressure, CVP–central venous pressure). Total vascular resistance (TVR), systemic vascular resistance (SVR) and pulmonary vascular resistance (PVR) were calculated: TVR = (MAP-CVP)/TF (MAP–mean arterial pressure, CVP–central venous pressure, TF–total flow), SVR = (MAP-CVP)/TFeff (MAP–mean arterial pressure, CVP–central venous pressure, TFeff–effective total flow), PVR = (PAMP-PCWP)/TF (PAMP–pulmonary artery mean pressure, PCWP–pulmonary capillary wedge pressure, TF–total flow).

### Data collection and statistics

All the data was real-time recorded and stored on a PC using ADI PowerLab ADC and LabChart Pro software (ADInstruments, New Zealand).

The data was analyzed using the Statistica software (StatSoft Inc., United States). Paired *t*-test or ANOVA were used as appropriate. Continuous variables are presented as mean ± SD. The *p*-value <0.05 was considered significant.

## Results

The protocol was performed on 7 animals in total. One of the animals suffered refractory ventricular fibrillation after initiating V-A ECMO support and was excluded from the study. Six animals were included into the data analysis, weighing 61.8 ± 6.2 kg.

### ECMO during maintained spontaneous cardiac output

#### Systemic haemodynamics

**FIGURE 3:**
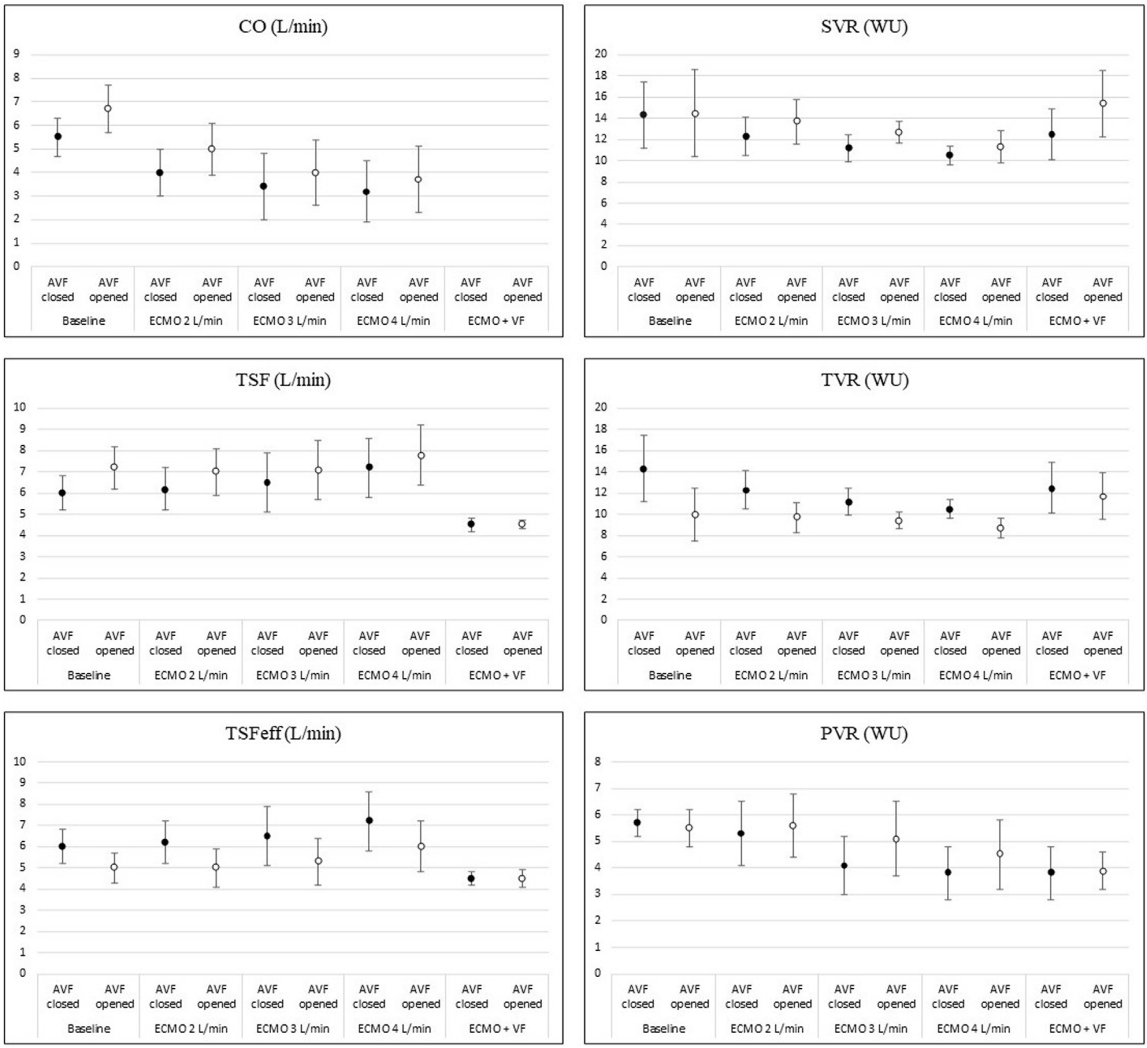
Changes of hemodynamic parameters during increasing ECMO flow and AVF opening. The picture shows changes in main hemodynamic parameters during increasing V-A ECMO flow and after opening the AVF. Details are discussed in the text.

Increasing the ECMO flow led to a rise in total systemic flow, although the cardiac output decreased. This also led to an increase in effective total flow. Details are displayed in Figure 3. The arteriovenous fistula blood flow was 2.2 ± 0.5 L/min at baseline (= 30.4 ± 4.1% of baseline CO). The AVF flow and the proportion of total flow consumed by the AVF gradually decreased with the increasing ECMO flow. AVF opening in each step led to an increase in total systemic flow due to the elevation of cardiac output. However, the effective systemic flow decreased after the AVF opening.

Systemic and pulmonary vascular resistance dropped with higher total systemic flow. Opening of the fistula decreased total vascular resistance in all steps. However, both the systemic and pulmonary vascular resistance increased. The effect of the AVF is displayed in Figure 3.

**FIGURE 4:**
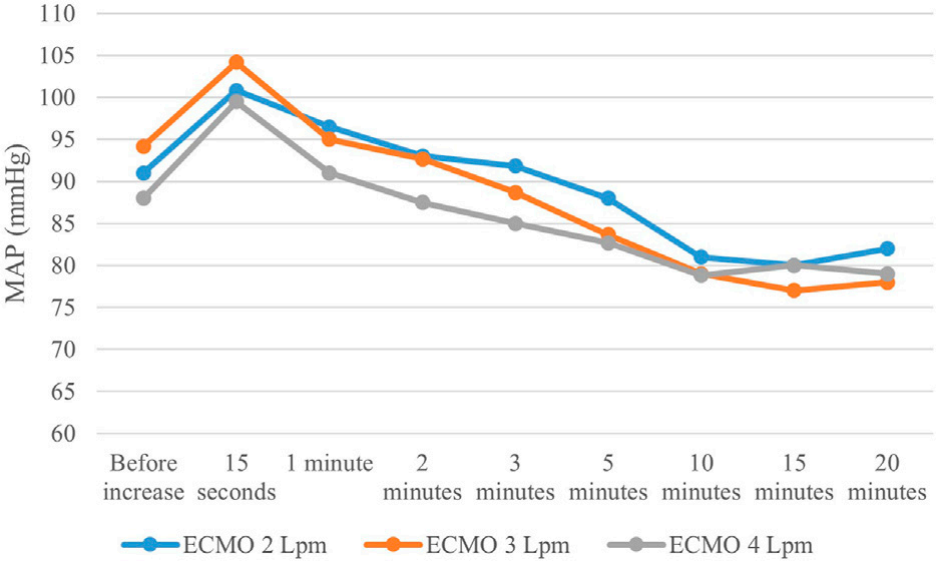
Mean arterial pressure changes after increasing ECMO flow. Mean arterial pressure showed the same pattern after each V-A ECMO flow increase–rapid rise followed by subsequential decrease, resulting in lower values than before change in ECMO flow.

Arterial pulse pressure gradually decreased at each step. Mean arterial pressure increased immediately after increasing ECMO flow, reaching its maximum approximately after 15 s; then gradually decreased and stabilized after 10 min, staying lower than before the ECMO flow increase. The course is displayed in Figure 4. Opening of the AVF further decreased mean arterial pressure in every step, and increased pulse pressure. Although the effect was lesser and unsignificant in 4 L/min step. The relation between AVF blood flow volume and mean arterial pressure was not significant. Pulmonary artery mean pressure increased after AVF opening. The results are summarized in Table 1.

**TABLE 1:** Effect of increasing ECMO flow and opening AVF on hemodynamic parameters and tissue perfusion.

Step	Baseline		ECMO 2 L/min		ECMO 3 L/min		ECMO 4 L/min	
Parameter	AVF closed	AVF opened	AVF closed	AVF opened	AVF closed	AVF opened	AVF closed	AVF opened
**CO (L/min)**	5.5 ± 0.8	6.7 ± 1.0*	4.0 ± 1.0**	5.0 ± 1.1*	3.4 ± 1.4**	4.0 ± 1.4*	3.2 ± 1.3**	3.7 ± 1.4
**Total systemic flow (L/min)**	6.0 ± 0.8	7.2 ± 1.0*	6.2 ± 1.0	7.0 ± 1.1*	6.5 ± 1.4	7.1 ± 1.4*	7.2 ± 1.4	7.8 ± 1.4
**Efective systemic flow (L/min)**	6.0 ± 0.8	5.0 ± 0.7*	6.2 ± 1.0	5.0 ± 0.9	6.5 ± 1.4	5.3 ± 1.1*	7.2 ± 1.4	6.0 ± 1.2*
**AVF flow (L/min)**		2.2 ± 0.5		2.0 ± 0.3		1.8 ± 0.3**		1.7 ± 0.3**
**% of flow through AVF**		30.4 ± 4.1		29.3 ± 3.1		26.2 ± 1.9**		22.6 ± 3.3**
**TVR (WU)**	14.3 ± 3.1	10.0 ± 2.5*	12.3 ± 1.8	9.7 ± 1.4*	11.2 ± 1.3	9.4 ± 0.8*	10.5 ± 0.9**	8.7 ± 0.9*
**SVR (WU)**	14.3 ± 3.1	14.5 ± 4.1	12.3 ± 1.8	13.7 ± 2.1*	11.2 ± 1.3	12.7 ± 1.0*	10.5 ± 0.9**	11.3 ± 1.5
**PVR (WU)**	5.7 ± 0.5	5.5 ± 0.7	5.3 ± 1.2	5.6 ± 1.2	4.1 ± 1.1**	5.1 ± 1.4*	3.8 ± 1.0**	4.5 ± 1.3*
**MAP (mmHg)**	89 ± 18	78 ± 23*	80 ± 17	74 ± 16*	76 ± 16**	72 ± 14	79 ± 11	73 ± 9*
**Pulse pressure (mmHg)**	37 ± 14	44 ± 14*	29 ± 9**	35 ± 11*	24 ± 11**	29 ± 13*	23 ± 9**	25 ± 11
**PAMP (mmHg)**	34 ± 4	39 ± 5*	32 ± 5	39 ± 5*	25 ± 4**	35 ± 5*	27 ± 5**	34 ± 6*
**CVP (mmHg)**	5.4 ± 1.5	6.2 ± 1.6	4.8 ± 1.6	6.4 ± 1.5*	4.2 ± 1.9**	6.2 ± 2.2*	4.4 ± 2.1	6.6 ± 2.3*
**SvO**_**2**_ **pulmonary artery (%)**	52 ± 9	60 ± 7*	59 ± 8**	56 ± 7	68 ± 11**	58 ± 8*	73 ± 8**	69 ± 12
**Coronary artery flow velocity (cm/s)**	17.8 ± 3.8	22.5 ± 4.9*	14.0 ± 5.5	16.1 ± 5.0	12.1 ± 4.8**	13.7 ± 5.3	11.6 ± 13.7**	13.7 ± 3.9*
**Carotid artery flow (mL/min)**	356 ± 150	267 ± 165*	412 ± 139	338 ± 127*	427 ± 142	375 ± 120*	467 ± 129**	436 ± 114
**Frontal lobe tissue flow (BPU)**	422 ± 189	336 ± 137	345 ± 155	311 ± 163	360 ± 160	348 ± 223	436 ± 224	442 ± 243
**Cerebral rSO**_**2**_ **(%)**	58 ± 7	53 ± 7*	63 ± 8	56 ± 6*	62 ± 7	59 ± 7*	63 ± 8	60 ± 7*
**Cerebral tissue pO**_**2**_ **(mmHg)**	15.9 ± 11.8	13.3 ± 10.2	14.1 ± 6.7	11.8 ± 5.7*	16.4 ± 7.3	13.9 ± 6.3*	14.8 ± 7.7	11.5 ± 5.0
**Tongue tissue flow (BPU)**	71 ± 17	59 ± 14*	70 ± 26	60 ± 13	85 ± 40	76 ± 32	94 ± 50*	83 ± 47

Statistically significant results are displayed in bold. Values are displayed as average ± standard deviation. *p*-value <0.05 was considered statistically significant. *—compared to step with closed AVF, **—compared to baseline.

CO, cardiac output; AVF, arteriovenous fistula; TVR, total vascular resistance; SVR, systemic vascular resistance; PVR, pulmonary vascular resistance; MAP, mean arterial pressure; PAMP, pulmonary artery mean pressure; CVP, central venous pressure; SvO_2_, saturation of mixed venous blood; rSO_2_—regional tissue oxygen saturation.

#### Tissue perfusion and oxygenation

The coronary blood flow velocity decreased with the increasing ECMO flow. Opening of the AVF increased the coronary artery blood flow velocity.

With closed AVF, the carotid artery blood flow continuously rose with the increasing total flow. Even during the hyper perfusion state, opening of the AVF significantly diminished the carotid artery flow in all steps except when ECMO flow was set to 4 L/min. Regional cerebral oxygen saturation measured by NIRS showed the same pattern as carotid artery flow, including significant drops in rSO_2_ after opening the AVF. However, invasively measured cerebral tissue blood microflow was not affected by the AVF. Tongue tissue flow dropped after each AVF opening; the effect of AVF lost significance in higher total systemic flow states. Cerebral tissue partial oxygen pressure stagnated while increasing total systemic flow and dropped after opening the AVF. The results are displayed in Table 1.

### ECMO during ventricular fibrillation

After the induction of VF, ECMO flow was set to maximal revolutions per minute (RPM). The ECMO flow reached 4.5 ± 0.3 L/min during VF with closed AVF and 4.5 ± 0.2 L/min with opened AVF (*p* = 0.72).

AVF flow during VF was substantially lower than at baseline 2.2 ± 0.5 L/min vs. 1.1 ± 0.2 Lpm, *p* = 0.007). However, the proportion of systemic flow stolen by the AVF did not change significantly (30% ± 4% in baseline vs. 24% ± 7% in VF, *p* = 0.08). Effective total flow during VF with closed fistula was lower than cardiac output in baseline (6.8 ± 0.8 vs. 4.5 ± 0.3 L/min, *p* = 0.003) and further decreased after AVF opening (to 3.4 ± 0.4 L/min, *p* = 0.00006 compared to VF with closed AVF). Opening of the AVF did not affect TVR (from 12.5 ± 2.4 WU to 11.7 ± 2.2 WU, *p* = 0.22), but increased SVR (from 12.5 ± 2.4 to 15.4 ± 3.1 WU; *p* = 0.001).

Carotid artery flow, cerebral rSO_2,_ tongue tissue flow and cerebral tissue blood microflow dropped after opening the AVF but the latter not significantly. Details are displayed in Table 2.

**TABLE 2:** Effect of AVF opening during ventricular fibrillation.

Parameter	Baseline - closed AVF	Baseline—opened AVF	VF on ECMO	VF on ECMO, AVF opened
**CO (L/min)**	5.5 ± 0.8	6.7 ± 1.0*		
**Total systemic flow (L/min)**	6.0 ± 0.8	7.2 ± 1.0*	4.5 ± 0.3	4.5 ± 0.2
**Effective systemic flow (L/min)**	6.0 ± 0.8	5.0 ± 0.7*	4.5 ± 0.3**	3.4 ± 0.4*
**AVF flow (L/min)**		2.2 ± 0.5		1.1 ± 0.2**
**% of flow through AVF**		30.4 ± 4.1		24.5 ± 6.5**
**TVR (WU)**	14.3 ± 3.1	10.0 ± 2.5*	12.5 ± 2.4	11.7 ± 2.2
**SVR (WU)**	14.3 ± 3.1	14.5 ± 4.1	12.5 ± 2.4	15.4 ± 3.1*
**PVR (WU)**	5.7 ± 0.5	5.5 ± 0.7	3.8 ± 1.0**	3.9 ± 0.7
**MAP (mmHg)**	89.3 ± 18.2	78 ± 23*	64.0 ± 9.7**	61.3 ± 9.9
**Pulse pressure (mmHg)**	37 ± 14	44 ± 14*		
**PAMP (mmHg)**	33.8 ± 3.9	39 ± 5*	16.8 ± 5.1**	17.8. ± 3.8
**CVP (mmHg)**	5.4 ± 1.5	6.2 ± 1.6	9.6 ± 1.1**	10.2 ± 2.3
**SvO2 pulmonary artery (%)**	52 ± 9	60 ± 7*	89.2. ± 4.9**	84.2 ± 10.3
**Coronary artery flow velocity (cm/s)**	17.8 ± 3.8	22.5 ± 4.9*	10.3 ± 3.9**	10.8 ± 3.8
**Carotid artery flow (mL/min)**	314 ± 121	267 ± 165*	380 ± 63	337 ± 64*
**Frontal lobe tissue flow (BPU)**	387 ± 139	336 ± 137	303 ± 88	279 ± 66
**Cerebral rSO2 (%)**	58.2 ± 7.0	53 ± 7*	53.6 ± 6.0**	51.4 ± 6.8*
**Cerebral tissue pO**_**2**_ **(mmHg)**	15.9 ± 11.8	13.3 ± 10.2	5.9 ± 3.3	5.1 ± 3.1
**Tongue tissue flow (BPU)**	71 ± 17	59 ± 14*	73 ± 31	60 ± 28*

Statistically significant results are displayed in bold. Values are displayed as average ± standard deviation. *p*-value <0.05 was considered statistically significant. *—compared to step with closed AVF, **—compared to baseline.

CO, cardiac output; AVF, arteriovenous fistula; VF, ventricular fibrillation; ECMO, extracorporeal membrane oxygenation; TVR, total vascular resistance; SVR, systemic vascular resistance; PVR, pulmonary vascular resistance; MAP, mean arterial pressure; PAMP, pulmonary artery mean pressure; CVP, central venous pressure; SvO_2_, saturation of mixed venous blood; rSO_2_, regional tissue oxygen saturation.

## Discussion

Our study confirmed a significant systemic steal caused by a high-flow AVF even during V-A ECMO support that affected mostly the cerebral circulation.

Opening of the arteriovenous fistula ads a low-resistance circuit to the circulation and leads to a drop in the total peripheral vascular resistance (resistance of systemic vascular beds + resistance of the AVF) (Guyton and Sagawa, 1961, Jr. 1970). However, systemic peripheral vascular resistance (resistance of the systemic vascular beds) increased after AVF opening as the organism tries to compensate the decrease of the perfusion pressure. As a result of the competition between the peripheral vascular resistance of various arterial beds and the AVF resistance, the AVF flow decreased in both absolute and relative values.

Decrease of coronary perfusion during V-A ECMO independent of arterial blood pressure has been observed in both animal and human studies (Smith, Whittlesey et al., 1989; Kato, Seo et al., 1996). Due to the position of ECMO cannula, coronary arteries are dominantly perfused by the remaining native cardiac output. Different factors could be responsible for this change–especially the decrease in cardiac output and subsequent compensation of lower metabolic demands of the myocardium or increase in coronary artery resistance caused by increased myocardial tissue pressure as suggested by Kato et al. (Kato, Seo et al., 1996). CO increase caused by AVF opening was, nevertheless, followed by immediate increase in coronary artery flow velocity. This supports the possibility that even during this unphysiological state, autoregulation of coronary blood flow by metabolic demands of the myocardium is maintained.

Mean arterial pressure slightly dropped with the increasing total flow. As depicted in Figure 4, MAP values slowly changed during each step. The explanation is not easy. We could only hypothesize that the rise of systemic blood flow increases the wall shear stress in the systemic arteries, which, in turn, leads to systemic arteriodilation. As the proportion of ECMO flow on systemic circulation rose, pulsatility of the arterial pressure curve decreased, as observed in previous studies (Chung, Shiloh et al., 2014; Hala, Mlcek et al., 2020).

In our experiment adding gradually increasing ECMO flow to a healthy circulation led to subsequential decrease of cardiac output, mean arterial pressure, pulmonary artery mean pressure, and both systemic and pulmonary vascular resistance. Venous cannula of the V-A ECMO drains blood from the inferior vena cava and thus decreases the right ventricular preload; the arterial cannula flushes blood into the aorta and increases afterload of the left ventricle (Distelmaier, Wiedemann et al., 2020). This was observed also by Popkova et al. (Popkova, Kuriscak et al., 2020).

The rise of carotid artery blood flow after increased ECMO flow added to spontaneous cardiac output has been already observed in previous animal experiments (Hala, Mlcek et al., 2020; Popkova, Kuriscak et al., 2020). In this study, we documented that even in this hyper-perfusion state the AVF kept stealing a significant proportion of carotid artery flow. Other authors suggested that cerebral autoregulation could be impaired during V-A ECMO (Short, Walker et al., 1993; Kazmi, Sivakumar et al., 2018). Our data rather shows that brain microcirculation remained unaffected by carotid artery flow decrease. Cerebral autoregulation is capable of maintaining sufficient cerebral blood flow during mean arterial pressure fluctuations between 60–160 mmHg (Armstead, 2016). The MAP remained within these limits during our experiments. We observed decreased cerebral rSO_2_ caused by high-flow arteriovenous fistula in both animal and human studies (Kovarova, Valerianova et al., 2021; Malik, Valerianova et al., 2021; Valerianova, Mlcek et al., 2022). Possible explanations of the detrimental effect of AVF on brain perfusion and oxygenation include systemic steal, flow competition or changes in vascular resistance and arterial blood pressure (Nickerson, Elkin et al., 1951; Amerling, Ronco et al., 2011).

Induction of ventricular fibrillation and subsequent loss of spontaneous cardiac output decreased the total flow and thus also the mean arterial pressure. Carotid artery flow and microcirculation in brain and tongue remained unaffected by this change; cerebral rSO_2_ decreased with lower systemic blood flow. Opening of the arteriovenous fistula in this setting did not lead to further arterial blood pressure drop, as it was compensated by immediate increase of systemic vascular resistance. The brain perfusion and oxygenation kept the same pattern as in the previous steps. Carotid artery blood flow volume and cerebral rSO_2_ decreased after opening the arteriovenous fistula, but microvascular flow decreased only non-significantly. However, we are aware that the low number of animals included in the study could affect the statistical significance.

The main limitation of the study is that it has been performed on otherwise healthy animals with normal heart and circulatory system. Humans (and animals) with end-stage renal disease are affected by many other possibly important complications, such as peripheral arterial narrowing. We believe the study at healthy pigs could bring some basic understanding of ECMO support in humans with a large arteriovenous fistula, but further studies are needed. Another limitation is that we simulated only the state of peripherally inserted ECMO cannulas. The hemodynamic effect of surgically created cardiopulmonary bypass with different positions of the cannulas can differ. We tried to minimize direct blood flow from ECMO to the AVF using different length of cannulas for ECMO circuit and AVF creation. However, we cannot fully exclude partial shunting of the blood straight to the fistula. On the other side, aortocaval fistula is used as a heart failure biomodel at rodents (Petrak, Havlenova et al., 2019).

## Conclusion

Our study showed that a large arteriovenous fistula can completely counteract the veno-arterial ECMO support unless maximal ECMO flow is applied. Cerebral blood flow and oxygenation are mainly compromised by the effect of the AVF.

Patients with high-flow AVFs used as hemodialysis access or with AVFs of other origin can get to critical health conditions requiring V-A ECMO support (cardiogenic shock, refractory cardiac arrest) or can undergo open heart surgery requiring cardiopulmonary bypass. Even though our study was performed on healthy animals with healthy hearts and circulatory system, we assume that changes in blood flow distribution and cerebral perfusion will follow the same pattern even in pathological states. Therefore, these observations could be of importance in patients with large arteriovenous fistulas in need of mechanical circulatory support. Johnson, 1970.

## Data Availability

The raw data supporting the conclusion of this article will be made available by the authors, without undue reservation.

## Abbreviations

AVF, arteriovenous fistula; CVP, central venous pressure; CO, cardiac output; Coeff, effective cardiac output; ECG, electrocardiogram; ECMO, extracorporeal membrane oxygenation; EtCO_2_, end-tidal carbon dioxide; HR, heart rate; i.m., intramuscular; i.v., intravenous; MAP, mean arterial pressure; PAMP, pulmonary artery mean pressure; pCO_2_, partial pressure of carbon dioxide; PCWP, pulmonary capillary wedge pressure; pO_2_, partial pressure of oxygen; PVR, pulmonary vascular resistance; RRR, replacement, reduction, refinement; rSO_2_, regional oxygen saturation; SvO_2_, venous oxygen saturation; SVR, systemic vascular resistance; TSF, total systemic flow; TSFeff, effective systemic flow; TVR, total vascular resistance; V-A ECMO, veno-arterial membrane oxygenation; VF, ventricular fibrillation; WU, Wood units.

## References

[B1] Amerling R. Ronco C. Kuhlman M. Winchester J. F. (2011). Arteriovenous fistula toxicity. Blood Purif. 31, 113–120. 10.1159/000322695 21228578

[B2] Armstead W. M. (2016). Cerebral blood flow autoregulation and dysautoregulation. Anesthesiol. Clin. 34, 465–477. 10.1016/j.anclin.2016.04.002 27521192 PMC4988341

[B3] Basile C. Lomonte C. (2018). The complex relationship among arteriovenous access, heart, and circulation. Semin. Dial. 31, 15–20. 10.1111/sdi.12652 28990213

[B4] Chung M. Shiloh A. L. Carlese A. (2014). Monitoring of the adult patient on venoarterial extracorporeal membrane oxygenation. ScientificWorldJournal 2014, 393258. 10.1155/2014/393258 24977195 PMC3998007

[B5] Distelmaier K. Wiedemann D. Lampichler K. Toth D. Galli L. Haberl T. (2020). Interdependence of VA-ECMO output, pulmonary congestion and outcome after cardiac surgery. Eur. J. Intern Med. 81, 67–70. 10.1016/j.ejim.2020.07.014 32736947

[B6] Guyton A. C. Sagawa K. (1961). Compensations of cardiac output and other circulatory functions in areflex dogs with large A-V fistulas. Am. J. Physiol. 200, 1157–1163. 10.1152/ajplegacy.1961.200.6.1157 13710075

[B7] Hala P. Mlček M. Ošťádal P. Popková M. Janák D. Bouček T. (2020). Increasing venoarterial extracorporeal membrane oxygenation flow puts higher demands on left ventricular work in a porcine model of chronic heart failure. J. Transl. Med. 18, 75. 10.1186/s12967-020-02250-x 32054495 PMC7017528

[B8] Johnson G. Blythe W. B. (1970). Hemodynamic effects of arteriovenous shunts used for hemodialysis. Ann. Surg. 171, 715–723. 10.1097/00000658-197005000-00010 4909703 PMC1396819

[B9] Kato J. Seo T. Ando H. Takagi H. Ito T. (1996). Coronary arterial perfusion during venoarterial extracorporeal membrane oxygenation. J. Thorac. Cardiovasc Surg. 111, 630–636. 10.1016/s0022-5223(96)70315-x 8601978

[B10] Kazmi S. O. Sivakumar S. Karakitsos D. Alharthy A. Lazaridis C. (2018). Cerebral pathophysiology in extracorporeal membrane oxygenation: Pitfalls in daily clinical management. Crit. Care Res. Pract. 2018, 3237810. 10.1155/2018/3237810 29744226 PMC5878897

[B11] Kovarova L. Valerianova A. Michna M. Malik J. (2021). Short-term manual compression of hemodialysis fistula leads to a rise in cerebral oxygenation. J. Vasc. Access 22, 90–93. 10.1177/1129729820924561 32489138

[B12] Malik J. Valerianova A. Tuka V. Trachta P. Bednarova V. Hruskova Z. (2021). The effect of high-flow arteriovenous fistulas on systemic haemodynamics and brain oxygenation. Esc. Heart Fail 8, 2165–2171. 10.1002/ehf2.13305 33755355 PMC8120398

[B13] McDonagh T. A. Metra M. Adamo M. Gardner R. S. Baumbach A. Böhm M. (2021). 2021 ESC Guidelines for the diagnosis and treatment of acute and chronic heart failure. Eur. Heart J. 42, 3599–3726. 10.1093/eurheartj/ehab368 34447992

[B14] Nickerson J. L. Elkin D. C. Warren J. V. (1951). The effect of temporary occlusion of arteriovenous fistulas on heart rate, stroke volume, and cardiac output. J. Clin. Invest. 30, 215–219. 10.1172/JCI102435 14814215 PMC436248

[B15] Petrak J. Havlenova T. Krijt M. Behounek M. Franekova J. Cervenka L. (2019). Myocardial iron homeostasis and hepcidin expression in a rat model of heart failure at different levels of dietary iron intake. Biochim. Biophys. Acta Gen. Subj. 1863 (4), 703–713. 10.1016/j.bbagen.2019.01.010 30677469

[B16] Popkova M. Kuriščák E. Hála P. Janák D. Tejkl L. Bělohlávek J. (2020). Increasing veno-arterial extracorporeal membrane oxygenation flow reduces electrical impedance of the lung regions in porcine acute heart failure. Physiol. Res. 69, 609–620. 10.33549/physiolres.934429 32584136 PMC8549886

[B17] Reddy Y. N. V. Melenovsky V. Redfield M. M. Nishimura R. A. Borlaug B. A. (2016). High-output heart failure: A 15-year experience. J. Am. Coll. Cardiol. 68, 473–482. 10.1016/j.jacc.2016.05.043 27470455

[B18] Short B. L. Walker L. K. Bender K. S. Traystman R. J. (1993). Impairment of cerebral autoregulation during extracorporeal membrane oxygenation in newborn lambs. Pediatr. Res. 33, 289–294. 10.1203/00006450-199303000-00018 8460067

[B19] Smith H. G. Whittlesey G. C. Kundu S. K. Salley S. O. Kuhns L. R. Chang C. H. (1989). Regional blood flow during extracorporeal membrane oxygenation in lambs. ASAIO Trans. 35, 657–660. 10.1097/00002480-198907000-00159 2597557

[B20] Soar J. Böttiger B. W. Carli P. Couper K. Deakin C. D. Djärv T. (2021). European resuscitation council guidelines 2021: Adult advanced life support. Resuscitation 161, 115–151. 10.1016/j.resuscitation.2021.02.010 33773825

[B21] Vahdatpour C. Collins D. Goldberg S. (2019). Cardiogenic shock. J. Am. Heart Assoc. 8, e011991. 10.1161/JAHA.119.011991 30947630 PMC6507212

[B22] Valerianova A. Mlcek M. Grus T. Malik J. Kittnar O. (2022). New porcine model of arteriovenous fistula documents increased coronary blood flow at the cost of brain perfusion. Front. Physiol. 13, 881658. 10.3389/fphys.2022.881658 35574433 PMC9091445

